# Self-Tolerance in a Minimal Model of the Idiotypic Network

**DOI:** 10.3389/fimmu.2014.00086

**Published:** 2014-03-10

**Authors:** Robert Schulz, Benjamin Werner, Ulrich Behn

**Affiliations:** ^1^Institute for Theoretical Physics, University of Leipzig, Leipzig, Germany

**Keywords:** idiotypic network, self-tolerance, control of autoreactive idiotypes, autoimmunity, bitstring model, mean-field theory

## Abstract

We consider the problem of self-tolerance in the frame of a minimalistic model of the idiotypic network. A node of this network represents a population of B-lymphocytes of the same idiotype, which is encoded by a bit string. The links of the network connect nodes with (nearly) complementary strings. The population of a node survives if the number of occupied neighbors is not too small and not too large. There is an influx of lymphocytes with random idiotype from the bone marrow. Previous investigations have shown that this system evolves toward highly organized architectures, where the nodes can be classified into groups according to their statistical properties. The building principles of these architectures can be analytically described and the statistical results of simulations agree very well with results of a modular mean-field theory. In this paper, we present simulation results for the case that one or several nodes, playing the role of self, are permanently occupied. These self nodes influence their linked neighbors, the autoreactive clones, but are themselves not affected by idiotypic interactions. We observe that the group structure of the architecture is very similar to the case without self antigen, but organized such that the neighbors of the self are only weakly occupied, thus providing self-tolerance. We also treat this situation in mean-field theory, which give results in good agreement with data from simulation. The model supports the view that autoreactive clones, which naturally occur also in healthy organisms are controlled by anti-idiotypic interactions, and could be helpful to understand network aspects of autoimmune disorders.

## 1 Introduction

B-lymphocytes express Y-shaped receptor molecules, antibodies, on their surface. These antibodies have specific binding sites which determine their idiotype. All receptors of a given B-cell have the same idiotype. B-cells with random idiotypes of remarkable diversity are produced in the bone marrow.

A B-cell is stimulated to proliferate if its receptors are cross-linked by complementary structures, unstimulated B-cells die. Proliferation occurs if the concentration of complementary structures is not too low or not too high, see e.g., Ref. (1). The latter condition refers to a steric hindrance for cross-linking if too many complementary molecules are around. Stimulating complementary structures can be found on foreign antigens and on other, so-called anti-idiotypic antibodies of complementary specificity. Thus B-lymphocytes can stimulate each other and form a functional network, the idiotypic network, as first proposed in Ref. (2), see also Ref. (3, 4).

The potential repertoire includes idiotypes that can recognize other complementary structures, e.g., on the active sites of enzymes, hormones, and neurotransmitters. Further, there are idiotypic interactions of B-lymphocytes with T-lymphocytes and between T-cells (5). Thus, the idiotypic network is not an autonomous entity of the adaptive immune system, but is coupled to many other networks.

Even for a hypothetical autonomous B-lymphocyte system, we have the requisites of evolution, random innovation, and selection. So the architecture of the idiotypic network can be conceived as the result of an evolution during the life time of an individual. In a revised version of the idiotypic network paradigm, the second generation idiotypic network (6–8), it was suggested that this architecture comprises a densely connected central part with autonomous dynamics and a hereto disconnected (or only sparsely connected) periphery. The periphery is able to clonal expansion in (an adaptive) response to external antigen, and since it is disconnected to the central part, the stimulation does not percolate through the network.

Already Jerne thought the idiotypic network to play an essential role in the control of autoreactive idiotypes (3). Today, the concept of idiotypic networks is still popular in the research on autoimmune diseases, both in theoretical studies and clinical context. Indeed, autoreactive antibodies are regularly found in healthy individuals though in low concentrations. Antibodies which escape other regulatory mechanisms can be controlled by the idiotypic network (9). Anti-idiotypic antibodies specific to potentially autoreactive clones are found in healthy individuals or in patients during remission, they are absent during periods of active autoimmune disease (10). Autoimmune diseases can be related to perturbations of the control of autoreactive clones (10–17), as for example in Myasthenia gravis, a well known B-cell associated autoimmune disease (18).

There are many alternative or complementary concepts to explain self-tolerance and a multitude of possible mechanisms to cause autoimmune diseases. It is of course beyond the scope of this paper to give an exhaustive review over this rapidly expanding field. We can list only a necessarily subjective selection of a few major concepts and mechanisms. Several theoretical concepts of self-nonself discrimination are presented in a topical issue of Seminars in Immunology (19), including the Two-signal theory (20), the Danger model (21), the context dependent tuning of T-cell antigen recognition (22), cf. also (23, 24), and the Immunological homunculus (25), cf. also (26, 27). Zinkernagel (28) emphasizes the importance of localization, dose, and time of antigens: antigen that does not reach secondary lymphoid organs in minimum doses or for sufficiently long times is immunologically ignored.

Regulatory T-cells have been identified to suppress a variety of immune responses and playing a crucial role in self-tolerance and in controlling the balance of T-helper cells such as Th1, Th2, and Th17 (29, 30). Various mechanisms how infections can trigger autoimmunity are reviewed in Ref. (31). Superantigens may cause a polyclonal T-cell response with an excessive cytokine release, which in turn can induce autoimmune disorders. Chronic tissue damage can, regardless of the initial stimulus, lead to a spreading of the specificity of the T-cell response (epitope spreading) including self-epitopes (32). More recently, epigenetic mechanisms which may cause a breakdown of immune tolerance have been identified in the context of several autoimmune diseases, for a review see Ref. (33), cf. also Ref. (34).

Recent progress in the understanding of autoimmune diseases is reviewed in a topical section of Current Opinion in Immunology edited by Wucherpfennig and Noel (35). The T-cell system and the B-cell system interact in various ways at different stages of an immune response and the distinction between B-cell mediated and T-cell mediated autoimmune disorders appears to erode (36). For T-independent features of B-cell response confer however (37). Also idiotype driven interactions exist between B-cells and T-cells, as reviewed in Ref. (38). Very recently, regulatory B-cells are brought into discussion (36, 39).

There are early attempts to model self-tolerance and autoimmunity mathematically within the network paradigm. We can distinguish papers which consider networks with predefined architecture from work, which studies the (ontogenic) evolution of the networks architecture.

In Ref. (40), based on experimental results (41), an idealized architecture of 26 clones was proposed, which comprises four groups of B-cell clones, a multi-affine group *A*, two mirror groups *B* and *C* with mutual coupling but no intra-group affinity, and a group *D* which couples with low affinity only to *A*. Based on this *ad hoc* architecture, a set of non-linear ordinary differential equations (ODEs) is proposed (42) that describes the continuous dynamics of B-cells and antibodies in the presence of self. The proliferation and maturation of by idiotypic interactions activated B-cells is modeled by the non-linear terms of the ODEs. Computer simulations of these ODEs reveal that the response of clones, which couple to self antigen depends on their connectivity to other clones of the network: the higher the connectivity the greater the degree of tolerance; poorly connected clones show unlimited growth.

In Ref. (43), an analytical theory for the dynamics of clones in the mirror groups *B* and *C*, which feel the mean-field exerted by the clones of group *A* that couple to self antigen is considered. The model describes a switching between tolerant and autoimmune states and reverse, induced by infection with external antigen.

Also a paper by Calenbuhr et al. (44) studies the behavior of idiotypic networks with predefined architecture in the presence of self. There, using a similar continuous dynamics as (42) the interaction between *N* clones of different idiotypes is determined by an *N* × *N* connectivity matrix (*N* = 2, … , 25) with entries zero and one. The maximum number of interactions *C* of a single clone with other clones is varied between 1 and *N* − 1 and open (chain like) and closed architectures are distinguished. The autonomous system shows oscillatory or chaotic behavior with parameter depending amplitudes. The response to a self-antigen depends on its concentration, and on the parameters of the autonomous system. The state of the system is called tolerant (safe) if the clones which couple to the self have low concentration, otherwise, for a large or even unbounded response, it is called dangerous. The study confirms that more densely connected networks tend to provide tolerant states.

Our work describing the *evolution* of the idiotypic network in the presence of self antigens is similar in spirit to previous work by De Boer and Perelson (45), Stewart and Varela (46), and Takumi and De Boer (47).

De Boer and Perelson (45) investigated a model which describes the population dynamics of antibodies and B-cells by a set of non-linear ODEs. The idiotype is modeled in a discrete shape space by bitstrings of length *L* (*L* = 32), two idiotypes match if the two aligned bitstrings are complementary in at least *T* adjacent positions (*T* is varied from 6 to 11, mainly *T* = 8) which mimics the presence of several idiotopes on an antibody with certain idiotype. For exactly *T* complementary positions an affinity of 0.1 is assigned, for more than *T* an affinity of 1. The stimulation of B-cells is described by a bell-shaped activation function, and the production of antibodies by stimulated B-cells by a gearing-up mechanism. There is an input of 10 new clones per day. They are incorporated in the network if at least one other clone is complementary. Clones with too high connectivity are suppressed. Simulations show that the network reaches a stationary regime where the idiotypes that are incorporated in the network are more similar than to be expected for a completely random choice. This gives an advantage because the incorporated B-cells feel a similar stimulating field and their (similar) antibodies do not form complexes. Among the clones which do not expand there are about 25% which have no sufficient stimulation. They are not incorporated in the network and can be considered as the clonal (peripheral) component of the immune system (similar to the singletons in our work, see below). Self antigen is also modeled by bitstrings. In high concentration it suppresses all clones which recognize the antigen, in stimulative concentrations (i.e., if their field is in the stimulating region of the bell-shaped activation function) it gives rise to unlimited self aggression. The authors mention that some of the self-reactive clones, especially those with a high connectivity, are controlled by overstimulation, clones with few connections escape the control.

Stewart and Varela (46) considered a model, which describes the presence or absence of clones of a given idiotype, not distinguishing B-cells and antibodies, using a discrete dynamics. A clone of idiotype *i* survives if it receives a stimulus σ*_i_* within an allowed window, σ*_L_* ≤ σ*_i_* ≤ σ*_U_*. If σ*_i_* is outside the window, the clone does not survive the next step of a parallel update. The stimulus an idiotype *i* receives from clones of complementary idiotype is calculated in a double-sheeted two-dimensional continuous shape space as $\sigma_{i}=\sum_{j}\;m_{ij}$ where $m_{ij}=\exp{\left\{-a_{ij}∕c\right\}}^{2}$. An idiotype is represented by a point on one of the sheets (say, the white one) while the perfectly complementary idiotype has the same coordinates on the other (black) sheet. *a_ij_* is the Euklidian distance of two points at different sheets and *c* is a characteristic distance below which idiotypic interactions are relevant. Simulations for periodic boundary conditions show that stationary patterns on the shape space emerge which consist of nested (concentric) black and white ellipses. They can be conceived as mirror groups where members of one group have only idiotypic interactions with the other group but not within their own group. Idiotypes disconnected from these groups (clonal components) only occur before saturation. The system needs a longer time to reach saturation as smaller *c* is. Self antigen is represented as points on the two-dimensional shape space. If located on the black (white) sheet it is incorporated in the black (white) elliptic lines. So if the bone marrow is able to produce idiotypes similar to the self, they buffer the self against aggressive autoimmunity.

Takumi and De Boer (47) investigated the evolution of a model network on a double-sheeted two-dimensional discrete shape space in the presence of self-epitopes. Self-reactive clones are deleted by hand assuming some not closer characterized self-tolerance process. Each idiotype has several determinants (idiotopes). New B-cell clones are generated randomly. The dynamics of B-cells is described by a system of ODEs with a log-bell-shaped activation function. A buffering term prohibits the explosion of the clone size, clones are removed if their size falls below an extinction threshold. Their main finding is that the network organizes such that most self-epitopes are embedded in an antibody repertoire of intermediate concentration. Without the explicit deletion of self-reactive clones the authors were unable to obtain robust self-tolerance.

The B-cell models mentioned above, describing the evolution of the network, have in common that they do not show an appropriate partitioning into network and disconnected fraction, and are not reliably stable when coupled to permanently present self antigen. Motivated by these drawbacks (48, 49) proposed to extend their previous models to include the cooperation with T-lymphocytes. Indeed, simulations of the ontogenic evolution of the network in the presence of self antigens (“founder” antigens) show that the system differentiates in several stages into two coexisting compartments, the central immune system that couples to and tolerates self antigens, and the peripheral immune system that could respond to “late” antigen. In the first stage, T-cells which become activated by the initial founding set of antigens, activate in turn B-cells. This continues until the B-cell repertoire is complete and the B-cells start to exert a regulatory feedback on the T-cells. In the second stage, the B-cells compete for T-cell help and their repertoire shrinks to B-cells of an idiotype, which directly recognize a T-cell receptor. After this, a single new antigen would elicit a response only of clones, which are not mounted to the network. However it turned out, that the peripheral system is too tolerant to a later antigen. This motivated (50) to further modify this model making the idiotypic connectivity an explicit function of time, and introducing a log-bell-shaped activation function also for the T-cells. Stewart and Coutinho (51) reviewed the state of modeling and the development of the paradigm, and critically mentioned the lack of experimental evidence supporting the physiological significance of idiotypic interactions between B-cell and T-cell receptors.

For more detailed reviews on the history of the paradigm, mathematical modeling, and new immunological and clinical developments the reader is referred to Ref. (52, 53). For very interesting personal accounts on the development of the network paradigm and the concept of immunological self, see Ref. (8, 54–57).

In the present paper, we consider a model of the idiotypic B-cell network proposed in Ref. (58) which describes the evolution toward complex, functional architectures. The model uses a discrete shape space spanned by bitstrings which represent idiotypes. The discrete dynamics describes presence or absence of idiotypic clones, which survive if their stimulus is within an allowed window. In a sense, the model combines the simplest features of the models previously proposed by De Boer and Perelson (45) and Stewart and Varela (46) and therefore can be considered as a minimal model.

The most interesting architecture emerging in this model comprises (i) densely linked core groups, (ii) peripheral groups without intra-group linking, (iii) groups of suppressed clones, and (iv) groups of singletons which potentially interact only with the suppressed clones. The expressed clones of the core and periphery groups build the actual network, the central part. The expressed clones of the singleton groups are not mounted to the network and can be considered as the peripheral or clonal component. This is clearly very close to the architecture envisaged in the concept of second generation idiotypic networks (6–8) and similar to the idealized *ad hoc* architecture of (40) but in our model these properties evolve from simple principles.

In the steady state, the size of these groups and their linking does not change with time. The groups are built from clones of different idiotypes, which have an individual dynamics but share certain statistical properties. The building principles of these architectures can be described analytically (59, 60), and the statistical properties can be calculated within a mean-field theory in good agreement with simulations (61).

Whereas the preceding work by Brede and Behn (58), Schmidtchen and Behn (59), Schmidtchen et al. (60), and Schmidtchen and Behn (61) considered the autonomous idiotypic network, i.e., the network of B-lymphocytes and their antibodies without foreign or self antigen, we investigate here the evolution of the idiotypic network, in the presence of self, toward an architecture where the expansion of autoreactive clones is controlled by idiotypic interactions. Self is modeled by permanently present idiotypes which influence the evolution of the network but are themselves not affected by idiotypic interactions. Our model avoids the above reviewed drawbacks of previous attempts, and the results clearly support the view that the idiotypic network is instrumental in the control of autoreactive clones.

The paper is organized as follows. In Section 2, we describe essential features of the model, its update rules, the general building principles which allow to understand the structural properties of the expressed networks architecture, and a tool which allows a real time identification of patterns in simulations. In Section 3, we sketch the derivation of the mean-field theory which allows to compute statistical properties if the structural properties of the pattern are known. In Section 4, we describe how the model should be modified in the presence of self. We report on simulations where the network in the presence of self evolves to an architecture such that the self is linked only to groups with very low population. Results of a modified mean-field theory are in good agreement with simulations. Finally, we give some conclusions and discuss problems for further research. There is a glossary where major key terms are briefly explained in a logical order.

## 2 The Model

In this paper, we consider a minimal model of the idiotypic network (58), which is a coarse simplification of the real biological system but retains most important features and reveals a surprising complexity. The model has only few parameters and allows an analytical understanding of many of its properties.

### 2.1 Potential repertoire and idiotypic interactions

We model the repertoire of all possible idiotypes and their interactions by an undirected network, where each node *v* of the network represents a distinct clone of B-lymphocytes of a given idiotype together with its antibodies. The idiotype is encoded by a bitstring of length *d* with entries 0 or 1. The number of different bitstrings 2*^d^* is the size of the potential repertoire. Note that the bitstrings are not thought to represent the genetic code or the sequence of amino acids but are meant as a caricature of the phenotype allowing an easy notion of complementarity. Interpreting the entries of the bitstrings as coordinates in a *d*-dimensional space each node can be conceived as a corner of a *d*-dimensional unit hypercube.

B-lymphocytes receive a stimulus to proliferate if their receptors are cross-linked by complementary structures, which can be situated on antigen but also on antibodies of complementary idiotype. We represent possible idiotypic interactions by links between nodes of nearly complementary idiotype. Assuming only perfect complementary receptor structures seems unrealistic and it appears reasonable to allow small variations. Therefore, two nodes *v* and *u* of our model are linked if their bitstrings are complementary allowing for up to *m* mismatches. We denote the undirected graph with 2*^d^* nodes labeled by bitstrings of length *d* and links between complementary nodes with up to *m* mismatches as base graph $G_{d}^{\left(m\right)}$. Each node of the graph is linked to $\kappa =\sum_{k=0}^{m}\;\left(\begin{array}{c} d\\ k \end{array}\right)$ nodes, which we will call the neighborhood of a node in the following. For example, consider in *d* = 12 the bitstring **1 1 1 1 1 1 1 1 1 1 1 1**, which is perfect complementary to the bitstring **0 0 0 0 0 0 0 0 0 0 0 0**. Replacing anyone of the zero’s in the latter by **1**, we obtain the 12 bitstrings which are complementary to the former except for one mismatch.

We only account whether an idiotypic clone is present or not and the corresponding node *v* is either occupied *n*(*v*) = 1 or empty *n*(*v*) = 0. The subgraph of occupied nodes, the expressed repertoire, with its links represents the expressed idiotypic network at a certain time. In the following subsection, we describe how the expressed idiotypic repertoire is generated.

### 2.2 Metadynamics and local dynamics

There is a continuous influx of new B-lymphocytes from the bone marrow. There, by somatic random reshuffling of the VDJ genes, which are responsible for the binding sites of the variable regions of an antibody, different idiotypes of an enormous diversity are generated. The potential repertoire is estimated to exceed the order of 10^10^ (62). We model this metadynamics by occupying, in each step of an iteration procedure, empty nodes of the expressed network with probability *p*.

The stimulation of a B lymphocyte to proliferate is a non-monotonous, log-bell-shaped, function of the concentration of complementary structures (63). The number of cross-linked receptors increases with the concentration of complementary structures. However, if their concentration is too high, cross-linking becomes less likely due to a steric hindrance and the stimulation decreases. An unstimulated B-lymphocyte dies. In our model an occupied node, i.e., a clone of a certain idiotype only survives if the number of its occupied neighbors is in an allowed window between two thresholds, *t_L_* and *t_U_*. The survival of a clone depends in a deterministic way on its local neighborhood in the shape space.

The dynamics is described in discrete time, the time step should be chosen such that an unstimulated cell will die within this time span and a stimulated cell can proliferate. The temporal evolution of the network is induced by the following update rules:

(i) Influx: occupy empty nodes with probability *p*.
(ii) Window rule: count the number of occupied neighbors *n*(∂*v*) of node *v*. If *n*(∂*v*) is outside the window [*t_L_*,*t_U_*], set the node *v* empty. This step is performed in parallel.
(iii) Iterate.

All three steps, the random global metadynamics, the deterministic local selection, and the iteration are of equal importance to describe an evolution of the network toward a complex architecture. Technically, our model can be categorized as a probabilistic cellular automaton, and also as a Boolean network, see Ref. (60) for a more detailed discussion.

**Figure 1:**
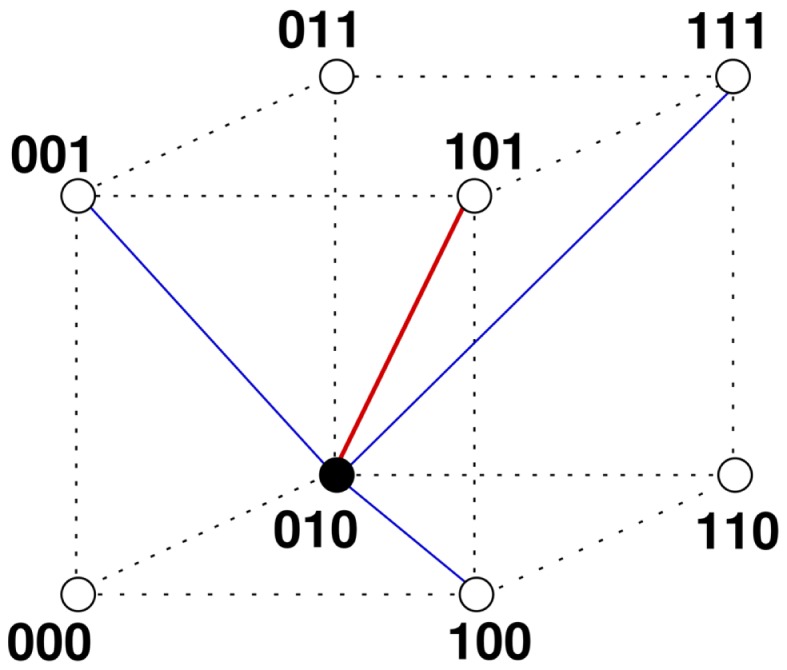
**Potential and expressed idiotypic repertoire**. The figure shows all nodes whose idiotypes are encoded by bitstrings of length *d* = 3. The nodes can be thought as the corners of a 3-dimensional cube. Also shown are the links of node 010 (filled circle) to the node with perfect complementary idiotype 101 (red line), and to the nodes with idiotypes 100, 001, and 111, which are complementary allowing for one mismatch (blue lines). The dotted lines are only to visualize the edges of the cube. The base graph $G_{d}^{\left(m\right)}$ for *d* = 3 and *m* = 1 consists of these 2*^d^* = 8 nodes and all links connecting complementary nodes allowing for up to one mismatch. It represents the potential idiotypic repertoire and the possible idiotypic interactions. The influx of B cells from the bone marrow is modeled occupying empty nodes with probability *p*. Assume that node 010 is occupied. If the neighboring nodes representing clones of complementary idiotypes are empty, it receives no stimulation and will die. It will also die if there are too many, say all 4, nodes of complementary idiotype occupied. So the node 010 survives the update only if the number of occupied neighbor nodes is within a window [*t_L_*,*t_U_*] = [1,3]. These steps, random influx and parallel application of the window rule for all nodes, are repeated and lead for larger dimensions *d* and appropriate parameters to a steady state with a complex architecture of the expressed idiotypic network, see text.

Figure 1 illustrates the construction of the base graph and the application of the update rules for the case *d* = 3, *m* = 1, [*t_L_, t_U_*] = [1,3].

Here, we report mainly on results for the following parameter setting, which is best investigated. The length of the bitstring is *d* = 12, then the network has 2^12^ = 4096 nodes. We allow *m* = 2 mismatches, which make the linking neither too sparse nor too dense, each node has κ = 79 neighbors. The lower threshold *t_L_* of the window rule has its minimal non-trivial value *t_L_* = 1: for survival of a clone the stimulation by at least one anti-idiotypic clone is required. The upper threshold of the window rule is chosen as *t_U_* = 10 that excludes very regular static patterns, which are in our context not interesting, for more details, see Ref. (60). Given these values, the influx probability *p* remains as main control parameter. In previous work (60, 61), we have studied a range for *p* from 0 to 0.1 and found that the architecture, which is of interest here evolves for *p* from 0.026 to 0.078. The results presented here explicitly are for *p* close to 0.078, where it is easier to initiate a reorganization of the pattern, but we have also studied a broader range of *p*. Simulations for longer bitstrings up to *d* = 22 have shown that many features are also found in larger networks and the major concepts of structural analysis are still applicable (64). The program code is implemented in C++. For small base graphs (*d* ≈ 12) optimization is not necessary. Larger base graphs (*d* ≈ 20) require optimization and parallel computing.

### 2.3 Building principles of the network architecture

Extensive simulations have shown that the network evolves, depending on the parameter choice, toward quasistationary states of possibly complex architecture (58). This architecture is characterized by groups of nodes that share statistical properties such as the mean occupation 〈*n*(*v*)〉 and the mean occupation of neighbors 〈*n*(∂*v*)〉. The mean occupation of the nodes, the groups, and of the whole base graph, i.e., the size of the expressed idiotypic repertoire, all are stationary – which implies homeostasis. Although, the mean occupation of a single node is stationary, its actual occupation switches in time between 0 and 1. These switches are induced by both the random influx from the bone marrow and the deterministic window rule. A statistical characteristics of this behavior is the mean life time, which is also stationary and the same for all nodes of a group.

There are general building principles of the network’s architecture which have been found by observing regularities in the bitstrings of nodes, which belong to the same group (59, 60). These principles make it possible to calculate the number of groups, their size, and the linkage between groups. Here, we only introduce the key terms and describe the essential results which are used in the following. For a deeper understanding of the derivation the reader should consult the original papers.

For a given architecture, the nodes can be classified according to their entries in the so-called determinant positions of the bitstrings. Different architectures have a different number *d_M_* ≤ *d* of determinant positions. The group *S*_1_ is defined as the set of all nodes with the same entries in all determinant positions, the entries in the non-determinant positions run through all $2^{d-d_{M}}$ possible combinations. Nodes in group *S*_2_ differ in one determinant position compared to nodes in *S*_1_, nodes in group *S*_3_ in two determinant positions, and so on. Consequently we have *d_M_* + 1 groups of size

(1) $$\begin{array}{l} |S_{g}|=2^{d-d_{M}}\left(\begin{array}{c} d_{M}\\ g-1 \end{array}\right) \end{array}$$

for *g* = 1, … , *d_M_* + 1 and we can immediately observe that groups *S_g_* and $S_{d_{M}+2-g}$ have the same size.

The whole architecture can be build from smaller units, so-called pattern modules. These modules are the corners of a *d_M_*-dimensional hypercube labeled by the determinant bits, together with the allowed links. Since the number of non-determinant bits is *d* − *d_M_*, the whole architecture is obtained by arranging 2*^d^*^−^*^d_M_^* identical pattern modules and adding the allowed links between the nodes of these modules.

Next, we discuss the linkage of our idiotypic network in a pattern with *d_M_* determinant bits on a base graph $G_{d}^{\left(m\right)}$. Each node in group *S_i_* has a fixed number *L_ij_* of links to nodes in group *S_j_*. The *L_ij_* are the elements of the link matrix 𝕃. Since the update rule counts the number of occupied neighbors and all nodes of a group have the same mean occupation these data are of obvious interest to formulate a mean-field theory. A careful analysis of the bitstrings which encode the nodes of groups *S_i_* and *S_j_* allows to derive an explicit expression (59, 60) which can be written as

(2) $$\begin{array}{llll} L_{ij} & =\sum_{k=0}^{m}\;\sum_{r=0}^{k}\;\left(\begin{array}{c} i-1\\ r \end{array}\right)\left(\begin{array}{c} d_{M}-i+1\\ j-1-r \end{array}\right)\; & & \;\\ & \;\times \left(\begin{array}{c} d-d_{M}\\ k+j-1-2r-\left(d_{M}-i+1\right) \end{array}\right). \end{array}$$

Given a pattern with *d_M_* determinant bits there are *d_M_* + 1 groups, therefore in equation (2) both *i* and *j* run from 1 to *d_M_* + 1. As every node has κ neighbors, the row sum of 𝕃 yields κ. Since $L_{ij}=L_{d_{M}+2-i,d_{M}+2-j}$ the link matrix is centrosymmetric, i.e., it fulfills the identity 𝕃*J* = *J*𝕃 where the exchange matrix *J* has entries 1 on the counterdiagonal and 0 elsewhere. 𝕃 describes a directed graph.

### 2.4 Real time pattern identification

In simulations, huge amounts of data are produced describing the occupation of each of the 2*^d^* nodes of the network in every single time step. An enormous, namely logarithmic reduction of information can be reached by introducing a center of mass vector **R** in dimension *d* which allows a real time identification of patterns and detection of pattern changes (60). Instead of monitoring 2*^d^* data per time step it is enough to observe the *d* components of *R*. The center of mass vector is defined as

(3) $$R=\frac{1}{n\left(G\right)}\sum_{v}\;n\left(v\right)r\left(v\right),$$

where the position vector **r**(*v*) of a node *v*, which is encoded by the bitstring $b_{d}b_{d-1}\cdots b_{1}$ with $b_{i}\in \left\{0,1\right\}$ has components *r_i_*(*v*) = 2**b_i_** − 1. *n*(G) is the total occupation of the basegraph G. By definition, for a symmetrically occupied base graph, we have **R** = **0**, a symmetry breaking pattern is easy to identify.

**Figure 2:**
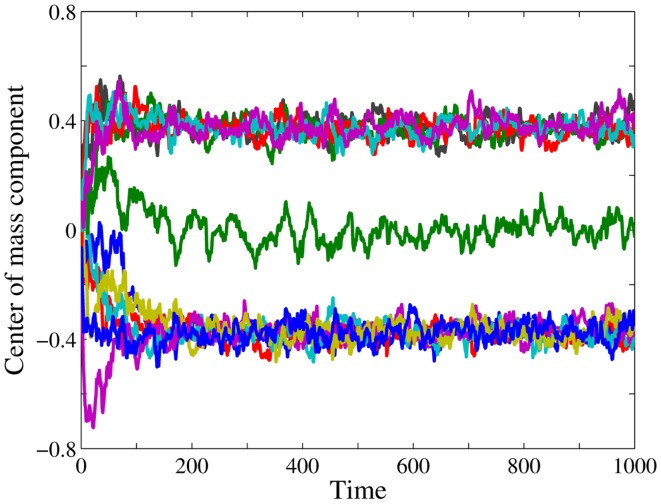
**Real time pattern identification**. Time series of the components of the center of mass vector **R** given by equation (3), here on the base graph $G_{12}^{\left(2\right)}$ for a window [*t_L_*,*t_U_*] = [1,10] and influx probability *p* = 0.074. Every color corresponds to one component of the center of mass vector. We start from an empty base graph, which is gradually occupied. A stationary state has evolved after about 200 time steps. The trajectory of *R*_2_ (green) fluctuates around zero. The corresponding bit position is non-determinant, see text. The trajectories of the five components *R*_7_, *R*_9_, *R*_10_, *R*_11_, *R*_12_ fluctuate around 0.4 and those of the 6 components *R*_1_, *R*_3_, *R*_4_, *R*_5_, *R*_6_, *R*_8_ around −0.4, The corresponding bit positions are determinant. Together there are 11 determinant bits, hence the dimension of the pattern module is *d_M_* = 11, and we can infer that the system has evolved toward a stationary 12-group architecture.

In Figure 2, we see the time series of the components of **R** for the evolution toward a stationary 12-group pattern. The trajectory of *R*_2_ fluctuates around zero. Since, the entries of non-determinant bits take for every group all possible values, and supposing that all nodes of a group are occupied with the same probability, the corresponding bit position can be identified as non-determinant. The trajectories of the five components *R*_7_, *R*_9_, *R*_10_, *R*_11_, *R*_12_ fluctuate around 0.4 and those of the 6 components *R*_1_, *R*_3_, *R*_4_, *R*_5_, *R*_6_, *R*_8_ around −0.4, The corresponding 11 bit positions are determinant. The dimension of the pattern module is *d_M_* = 11, thus we have a 12-group architecture. Furthermore, we can readily identify the determinant bits of the group *S*_1_. As explained below, *S*_1_ is a peripheral group with high occupation. The observation that five components of **R** fluctuate around a positive value indicates that the five determinant bits at the corresponding positions should have entry 1, and the other six should have entry 0. Thus, the nodes of group *S*_1_ have a bitstring **1 1 1 1 0 1 0 0 0 0 ***⋅*** 0**, where the ***⋅*** represents the only non-determinant bit. The determinant bits of *S*_12_ are complementary, and also nodes of the other groups are easily identified knowing their bitstrings. The reader who is interested in further technical details should consult (60).

The procedure is fast, robust against defects of patterns, and allows to identify pattern changes. Needless to say, the method hinges by construction on the encoding of the idiotype by bitstrings, which is only a gross caricature of the phenotype. Here, we use this tool to characterize the behavior of the network if several nodes become permanently occupied to mimic the presence of self.

## 3 Mean-Field Theory

Once established, an architecture, characterized by the number of groups, their size, and their linking remains stationary for long periods of time and over some range of the main control parameter *p*. As shortly sketched above, for most architectures found in simulations their characteristics can be computed knowing the number of determinant bits *d_M_*, which can be inferred from the time series of the center of mass coordinates.

The statistical properties of the nodes, which belong to the same group, such as the mean occupation and the mean life time, depend however on the actual value of *p*. They can be calculated (61) adopting the concept of mean-field theories, which was developed in statistical physics to describe phase transitions and has been transferred to many other problems in different fields. The main argument goes as follows.

The window rule (ii) for update of the occupation of a node counts only the total of the occupied neighbors. All nodes of a group have the same number of neighbors in the other groups given by the elements of the link matrix. The occupation of these neighbors typically fluctuates in time, and if they are many, it appears natural to replace the actual occupation of the neighbors by the average occupation. This works the better, the more neighbors are involved. In this view, a node feels only the different mean-fields, the modular mean-fields, exerted by the occupied neighboring nodes belonging to the different groups.

We now shortly describe the derivation in a more formal way to make the modifications understandable which are necessary when modeling the presence of self. Consider an architecture, which can be described by pattern modules of dimension *d_M_*. Then, we have *d_M_* + 1 groups of nodes *S_g_* which share the mean occupation $\langle n\left(v_{g}\right)\rangle =n_{g}$ where *v_g_* ∈ *S_g_*, their linking is described by the link matrix 𝕃. The set of mean occupations $n={\left(n_{1},\cdots \;,n_{d_{M}+1}\right)}^{\text{T}}$ defines the state of the network in the reduced mean-field description at a certain time. Application of the update rules to **n** leads to a new state ${\text{n}}^{\prime }$ given by

(4) $$n^{\prime }=f\left(n\right),$$

where the non-linear function **f** depends on the update rules and on the pattern we want to describe. We know that a node *v_g_* of group *S_g_* has *L_g1_* neighbors in *S_l_*. If the mean occupation in *S_l_* is *n_l_*, the new mean occupation after the influx with probability *p* is ${\tilde{n}}_{l}=n_{l}+p\left(1-n_{l}\right)$. The probability that *k_l_* nodes of the neighborhood in *S_l_* are occupied after the influx is

(5) $$\left(\begin{array}{c} L_{gl}\\ k_{l} \end{array}\right)\;{\tilde{n}}_{l}^{\;k_{l}}{\left(1-{\tilde{n}}_{l}\right)}^{L_{gl}-k_{l}}.$$

Supposing that the groups are independent, the probability that for a micro-configuration with fixed *k_l_, l* = 1, … , *d_M_* + 1, a total of $\sum_{l=1}^{d_{M}+1}\;k_{l}$ neighbors is occupied is simply the product of factors (5) for each group. Summing over all micro-configurations and taking into account the window rule leads to

(6) $$\begin{array}{l} {\left[\sum_{k_{l}=0}^{L_{gl}}\right]}_{l=1}^{d_{M}+1}𝟙\left(t_{L}\leq \sum_{l=1}^{d_{M+1}}\;k_{l}\leq t_{U}\right)\prod_{l=1}^{d_{M+1}}\;\left(\begin{array}{c} L_{gl}\\ k_{l} \end{array}\right){\tilde{n}}_{l}^{k_{l}}{\left(1-{\tilde{n}}_{l}\right)}^{L_{gl}-k_{l}}, \end{array}$$

where the indicator function 𝟙 (⋅) gives one, when the window rule in the parameters is fulfilled, otherwise zero. The last result should be multiplied with the mean occupation of a node of the considered group after the influx ${\tilde{n}}_{g}=n_{g}+p\left(1-n_{g}\right)$ which gives

(7) $$\begin{array}{llll} {n^{\prime }}_{g} & ={\tilde{n}}_{g}{\left[\sum_{k_{l}=0}^{L_{gl}}\;\right]}_{l=1}^{d_{M}+1}𝟙\left(t_{L}\leq \sum_{l=1}^{d_{M+1}}\;k_{l}\leq t_{U}\right)\; & & \;\\ & \;\times \prod_{l=1}^{d_{M+1}}\left(\begin{array}{c} L_{gl}\\ k_{l} \end{array}\right){\tilde{n}}_{l}^{k_{l}}{\left(1-{\tilde{n}}_{l}\right)}^{L_{gl}-k_{l}}. \end{array}$$

Iterating equation (7), for *g* = 1, … , *d_M_* + 1, the ${\text{n}}^{\prime }$ converge to a fixed point n*. Since **f**(**n**) is a non-linear function, several fixed points may exist. As a thumb rule, initial values close to the stationary average values seen in simulations are in the basin of attraction of fixed points of equation (7), which reproduce the simulation results. There may exist other fixed points, which were not found in simulations, for details see Ref. (61).

## 4 Idiotypic Network and Self

**Figure 3:**
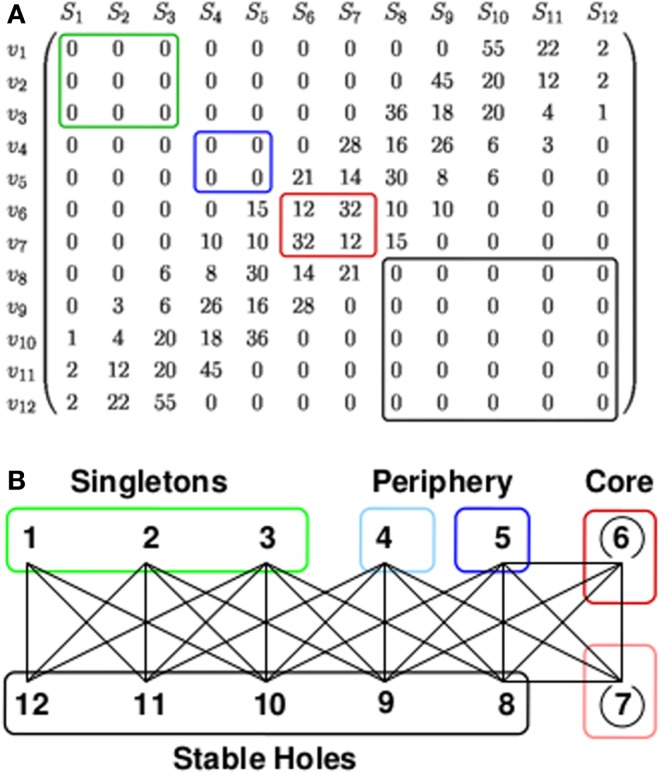
**12-group architecture**. **(A)** The entries *L_ij_* of the link matrix, given by equation (2), show the number of neighbors a node *v_i_* of group *S_i_* has in group *S_j_*. For example, the first row of the matrix tells that every node of the singleton group *S*_1_ (denoted by *v*_1_) is linked only to nodes of the hole groups *S*_10_, *S*_11_, and *S*_12_, namely to 55, 22, and 2 nodes, respectively. Only nodes of *S*_6_ and *S*_7_ (red box) have links to other nodes of the own group. **(B)** The architecture generated by this link matrix together with a phenomenological classification into singletons (green), periphery (blue), core (red), and stable holes (black). The lines symbolize the existence of links between nodes of the connected groups, i.e., possible idiotypic interactions between the corresponding clones. The number of links which a node in *S_i_* has to nodes in *S_j_* is given by the element *L_ij_* of the link matrix shown in **(A)**. The weakly occupied core groups have links within the own groups (open circles). The periphery groups are highly occupied and couple to the core and to the group of stable holes. The group of singletons is highly occupied and couples only to the stable holes. This architecture evolves on the base graph $G_{12}^{\left(2\right)}$ for a window [*t_L_*,*t_U_*] = [1,10] and a range of the influx probability *p* from 0.026 to 0.078. See also Glossary.

The 12-group architecture is of particular interest, as it strongly resembles the central and peripheral parts of the second generation idiotypic network. A scheme of these architecture is given in Figure 3. The 12-group architecture evolves on the base graph $G_{12}^{\left(2\right)}$ for [*t_L_*,*t_U_*] = [1,10] and a range of *p* from 0.026 to 0.078. The groups comprise two self coupled core groups, two peripheral groups, which couple only to the core and five groups of stable holes. Stable holes are typically unoccupied since their occupied neighbors exceed *t_U_*. Finally, there are three groups of singletons which are neighbored only by stable holes. Nodes of the singleton groups have an average occupation of 0.2–0.8, nodes of the periphery groups have 0.4–0.8 depending on *p*. The average occupation of the densely linked core groups is kept below 0.07, and the holes are almost empty, for details see Figure 8 in Ref. (61). Note that the singletons have no links to the connected part of the occupied network, which is built of the core and periphery groups. In terms of the second generation idiotypic networks, core, and periphery groups form the central part. The singletons, disconnected from the central part, form the clonal component (the peripheral part) of the second generation network.

The simplest possible way we can imagine to mimic the presence of self is to permanently occupy one or several nodes of the base graph and investigate their influence on the network architecture. The self nodes contribute to the number of occupied neighbors counted in the window rule but are themselves not affected by idiotypic interactions. The window rule does not apply to self nodes. We performed two types of computer experiments, inserting permanently occupied nodes in a fully developed 12-group architecture and monitoring the induced changes, or in an empty base graph and observing from scratch the evolution of the networks architecture.

Naturally, the influence of the permanently occupied nodes increases with their number. Their impact also depends on the influx rate *p* since the 12-group architecture becomes unstable for *p* ⪆ 0.08. Inserting self nodes in the established architecture, close to this threshold the strongest impact is to be expected.

### 4.1 Simulations

We performed extensive simulations for different protocols. Here, we describe only few most instructive cases.

**Figure 4:**
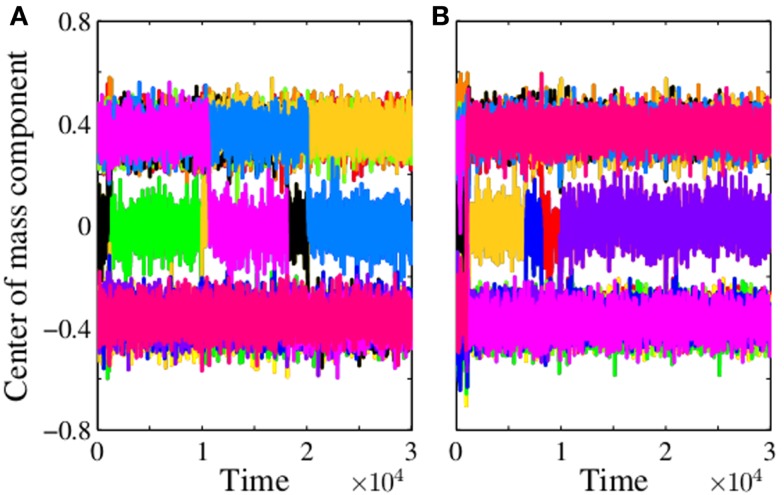
**Reorganization of the 12-group architecture with self**. The figures displays the time series of the components of the center of mass vector obtained from simulations for an influx probability *p* = 0.076 when at *t* = 0 one node **(A)** or 10 nodes **(B)** of the hole group *S*_10_ are permanently occupied. Each of the 12 components is drawn with a different color. They are plotted one after another, only the last printed color is visible. The trajectories mainly fluctuate around ±0.4 and zero. Jumps between these values indicate changes of the determinant bits associated with a reorganization of the architecture. For one self node **(A)** we see five jumps and for *t* ⪆ 2 × 10^4^ a stationary state is reached. For 10 self nodes **(B)**, after a few jumps, the stationary state is already reached for *t* ⪆ 10^4^, obviously the impact of 10 self nodes is stronger than the impact of one. A closer look at the data (not discussed here) shows that the new stationary pattern has indeed a 12-group architecture where the self node in case **(A)** belongs to periphery group *S*_5_ and in case **(B)** four self nodes belong to the singleton group *S*_3_ and the remaining six self nodes belong to the periphery group*S*_5_.

We permanently occupy one node of the hole group *S*_10_ of an established 12-group pattern for *p* = 0.076, i.e., close but below the upper threshold of stability of the pattern. The hole groups have many occupied neighbors and a self node staying there would be subject of a heavy autoimmune response. After few iterations, the former stable pattern destabilizes under the presence of the self and collapses. Thereafter, a new 12-group architecture evolves where the self node is now located in a group with only weakly occupied neighbors, which could be one of the singleton or periphery groups. Figure 4A shows the time series of the center of mass components for an example where the permanent occupied node (the self) is after a reorganization of the architecture finally in the periphery group *S*_5_.

If we permanently occupy more than one node the scenario is similar. Figure 4B shows an example where we have permanently occupied 10 nodes of the hole group *S*_10_ of an established 12-group pattern for *p* = 0.076. The reorganization of the architecture is faster and in the new steady state the self nodes are found in singleton and periphery groups.

We also performed simulations where for a stationary 12-group pattern all members of the hole group *S*_10_ are permanently occupied. After reorganization of the architecture, in the steady state all self nodes belong to singletons and periphery groups and are never seen in a core or a hole group. If we start from an established 12-group pattern and permanently occupy one of the singletons or periphery groups this state will be stable for very long periods of time.

Starting from an empty base graph with several permanently occupied nodes, one observes that the architecture evolves from the very beginning such that the self nodes have only weakly occupied neighbors and thus are tolerated. This evolution from scratch toward a tolerant architecture occurs for a much broader range of *p* than the reorganization of an established architecture.

### 4.2 Mean-field theory with self

It is possible to modify the mean-field theory to describe a stationary architecture in the presence of self. We thus can describe situations where in an established pattern nodes are permanently occupied and the impact is so small that no reorganization sets in. If the impact is strong enough that a reorganization occurs and a new steady state emerges, we also can describe statistical properties of this steady state, such as the mean occupation of nodes and its neighbors, provided that we know its architecture.

We first consider one permanently occupied node of group *S_s_*. It is linked to nodes of group S*_g_* if *L_sg_* > 0. The group *S_g_* contains *L_sg_* nodes that see the self. For these nodes we should modify the mean-field mapping, equation (7). The node of *S_s_* which is permanently occupied should be exempted from the combinatorics of possible and allowed micro-configurations. Thus, we need to replace *L_gs_* by *L_gs_ −* 1. Observe that $\left(\begin{array}{c} L_{gs}-1\\ k_{s} \end{array}\right)$ in the modified equation (7) is zero if *L_gs_ −* 1 is smaller than zero or *k_s_*. To account for the permanently occupied self node, we should decrease both thresholds of the window condition by 1. For the $|S_{g}|-L_{sg}$ nodes of *S_g_* which do not see the self node, the mapping is not modified. For example, for an influx with *p* = 0.07 and one permanently occupied node in a hole group or in a core group, 〈*n*(∂*v*)〉 increases by about 1 and 〈*n*(*v*)〉 decreases by about 20% if *v* is linked to the self node. The mean-field theory agrees with the simulation within 3–5%.

The case that all nodes of a group *S_s_* are permanently occupied is even simpler because all nodes in group *S_g_* see the same number *L_sg_* of self nodes. We only have to modify the window condition decreasing both thresholds by *L_sg_*. Note that if *t_U_* − *L_sg_* < 0 the modified window condition cannot be fulfilled and the indicator function 𝟙 (⋅) in the modified equation (7) returns 0. Table 1 gives a detailed comparison of simulation and mean-field theory for the case that all 110 nodes of the singleton group *S*_10_, cf. equation (1) for *d* = 12, *d_M_* = 11, are occupied for *p* = 0.074.

**Table 1:** **12-Group architecture with self after reorganization**.

Group	〈*n*(*v*)〉				〈*n*(∂*v*)〉			
	Simulation		MFT		Simulation		MFT	
*S*_1_	0.0		0.0		71.75	(54.01)	71.40	(53.99)
*S*_2_	0.0		0.0		60.34	(53.86)	60.29	(53.96)
*S*_3_	0.0		0.0		59.62	(53.50)	59.85	(53.52)
*S*_4_	0.0		0.0		36.70	(34.86)	36.62	(34.72)
*S*_5_	0.0		0.0		31.5	(29.62)	31.63	(29.73)
*S*_6_	0.002	(0.001)	0.0		13.52	(13.53)	13.63	(13.63)
*S*_7_	0.01		0.003		10.12	(10.09)	10.10
*S*_8_	0.677	(0.661)	0.6708		0.15	(0.14)	0.07
*S*_9_	0.706	(0.681)	0.6827		0.025	(0.018)	0.01
*S*_10_	1.0	(0.685)	1.0	(0.685)	0.02	(0.0)	0.0
*S*_11_	0.685	(0.684)	0.6835	(0.685)	0.001	(0.0)	0.0
*S*_12_	0.685	(0.682)	0.6835	(0.685)	0.001	(0.0)	0.0

**Figure 5:**
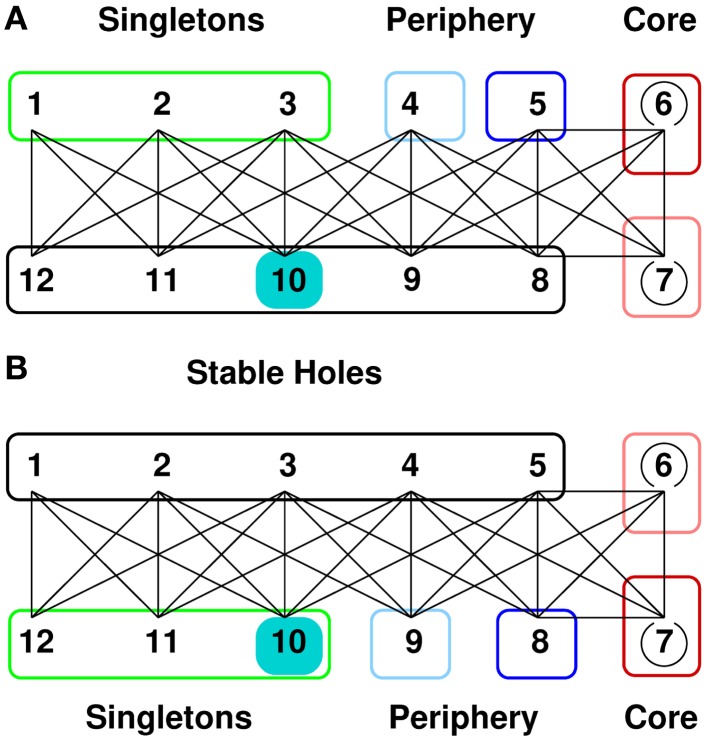
**12-group architecture with self**. **(A)** We permanently occupy one of the hole groups, group 10 (cyan), thus mimicking the permanent presence of self. This state is not favorable since the self couples to singletons and periphery, which have a high occupation. **(B)** Letting the thus prepared system evolve, it soon reaches a new steady state, still a 12-group architecture, but organized such that the self now belongs to the singletons and thus couples only to the almost empty stable holes. The self-recognizing idiotypes are controlled by the network, thus providing self-tolerance.

*The 110 nodes of the singleton group *S*_10_ are permanently occupied to mimic the presence of self antigen, see Figure 5B. The table shows the mean occupation 〈*n*(*v*)〉 and the mean occupation of neighbors 〈*n*(∂*v*)〉 for all groups as obtained for *p* = 0.074 from simulations and from mean-field theory (MFT) with a *d*_*M*_ = 11 module. When deviating, the data for the case without self are given in parentheses. The groups *S*_1_, … , *S*_5_ have direct neighbors in *S*_10_, where *S*_1_ has the most ones. Therefore, the change in 〈*n*(∂*v*)〉 due to self is largest for *S*_1_. Results from simulation and mean-field theory are in good agreement. The simulation data are obtained as follows. We first computed the temporal average of each node’s occupation from 30,000 time steps. Then the mean of these data over all nodes of the same group is calculated. The variance of the mean over the group members is of the order 10^−3^*.

For *N_s_* self nodes with 1 < *N_s_* < |*S_s_*| the modification is also possible but more intricate and will not be reported here.

**Figure 6:**
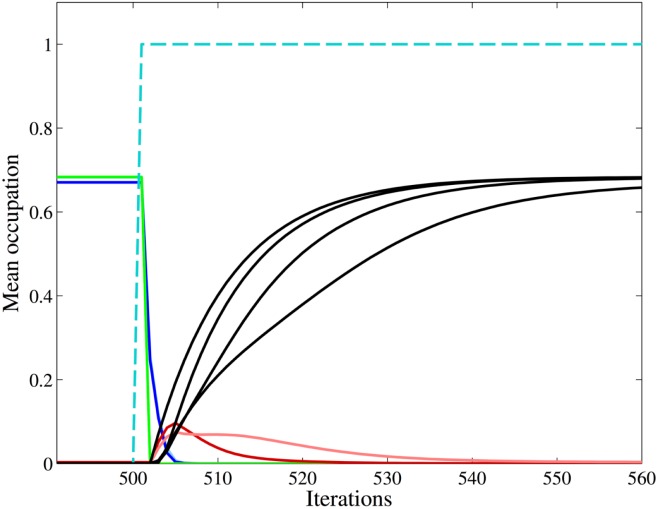
**Mean-field theory of a 12-group architecture with self**. The figure shows the time series of the mean occupation per node of the 12 groups as obtained from iterating equation (7) for an influx probability *p* = 0.074. We start with the autonomous system in the steady state where singletons (green) and periphery (blue) have a mean occupation per node 〈*n*〉 ≈ 0.68, whereas core (red) and stable holes (black) have 〈*n*〉 ≈ 0. The color code is the same as in Figure 5A. At some arbitrary iteration step (here 500), we permanently occupy the 110 nodes of the hole group *S*_10_ (dashed cyan line). The fixed point of equation (7) looses its stability and a new mirrored architecture emerges where the permanently occupied nodes, the self, now belong to the singletons, which have neighbors only in the empty hole groups, cf. Figure 5B. The occupation of the previous singleton (green line) and periphery (blue line) groups drops down to almost zero, whereas the previous hole groups (black lines) become occupied as typical for singletons and periphery. (The light blue line of the periphery group *S*_4_ is not visible here since it is covered by the green line up to iteration step 500, and thereafter by the blue line.) After an temporary increase the core groups (red lines) return to its previous occupation. For a detailed comparison of steady state results of mean-field theory and simulation see Table 1.

Encouraged by the good quantitative agreement between the steady states obtained in simulations and mean-field theory, we also looked at the time series of **n** generated by the mean-field mapping for a *d_M_* = 11 pattern at *p* = 0.074 to see the effect induced by permanently occupying a group of nodes. We start with the fixed point n*, which describes a 12-group pattern where the groups are ordered as in Figure 5A. In the steady state, at an arbitrary iteration step, we permanently occupy the hole group *S*_10_. The time series, cf. Figure 6, shows that this state immediately destabilizes and that a reorganization sets in. The pattern converges to a new state where the self belongs to the new singleton group *S*_10_. These singletons have only neighbors in the new unoccupied hole groups, see Figure 5B. The network controls the expansion of the autoreactive idiotypes in the hole groups – thus providing self-tolerance. Analogous results (not shown here) are obtained if we permanently occupy the hole group *S*_9_, after reorganization group *S*_9_ is a periphery group coupling only to the holes and to the weakly occupied core.

We note in this context that due to the centrosymmetry of the link matrix of the autonomous network without self, given a fixed point $n^{*}={\left(n_{1}^{*},n_{2}^{*},\ldots ,n_{d_{M}+1}^{*}\right)}^{T}$, there exists always a mirrored fixed point $n_{\mathrm{mirror}}^{*}={\left(n_{d_{M}+1}^{*},\ldots ,n_{2}^{*},n_{1}^{*}\right)}^{T}$. Obviously this symmetry is broken if self is present.

## 5 Conclusion and Outlook

We have extended a minimal model of the idiotypic network (58, 60, 61) to study the evolution of the network in the presence of self. Self is represented by permanently occupied nodes of certain idiotypes. These self nodes can stimulate autoreactive clones and thus influence the evolution of the network but are themselves not affected by the idiotypic interactions. We report on simulation results for the case that the self nodes are permanently occupied already at the initial state. Then, the network evolves toward an architecture where the permanently occupied self nodes are incorporated into groups of nodes which have, in a sense, a similar idiotype. These groups can idiotypically interact only with other groups that are either completely suppressed by the network (stable holes) or only weakly occupied. The network controls the expansion of self-reactive clones thus providing self-tolerance.

We also studied the response of a network with an already established architecture to a sudden appearance of self antigen. Nodes of the hole groups were permanently occupied, which is most unfavorable since these groups are linked to highly occupied clones. Provided that the influx from the bone marrow is sufficiently high the network reorganizes its architecture such that in the end the self nodes belong to groups, which have only empty ore weakly occupied neighbors, as in the previous case.

For the simplest cases that only one node or all nodes of a group are permanently occupied, we have modified the mean-field theory and found good agreement of analytical and simulation results.

As discussed in the introduction to some extent, there are preceding attempts in the literature, which aim in the same direction but were not really satisfying. Our results strongly support the view that idiotypic interactions can be instrumental in the control of autoreactive clones.

The network in the presence of self has been previously studied by one of us in simulations for one self node on the base graph $G_{12}^{\left(3\right)}$ with weighted links. The weights were given according to the number of mismatches of the linked nodes and the window condition was modified accordingly. The patterns are slightly easier to destabilize which explains why the phenomenon of self-tolerance was first observed in that version of the model (65).

Further studies should systematically explore the system’s behavior for other protocols, e.g., for arbitrary numbers of self nodes possibly distributed over the whole base graph, desirably in both simulations and an accordingly extended mean-field approach.

It is of obvious interest to investigate in the frame of the model possible reasons for failure of self-tolerance. Transitions from a healthy self-tolerant state to an autoimmune state by a perturbation, possibly an ordinary infection, of the clones that control the autoreactive idiotypes should be considered, together with the reverse phenomenon of ’spontaneous’ remission from an autoimmune to a healthy state. Therapeutic strategies adopting the network paradigm (66), which consist in stimulating the protective clones that control the autoreactive clones, instead of applying immunosuppressive drugs, could be modeled.

To study age induced effects, it would be very interesting to consider an influx rate *p* from the bone marrow, which decreases over the lifespan of an individual. The architecture which controls autoreactive clones is found for a certain range of *p*. However, if we suddenly stop the influx at all, the group of singletons, which is only sustained by the influx would be depopulated, and the other, connected part of the network would, in a sense, freeze. Since the singletons play an important part in controlling the autoreactive clones this should have consequences for maintaining self-tolerance. A small influx outside the range mentioned above would lead to less complex architectures which may be not functional.

The renewal rate of the expressed idiotypic repertoire is certainly relevant in the physiological context. Therefore, it would be interesting to determine this characteristics in the frame of our model. It is of course related to the influx from the bone marrow but also depending on the population dynamics of the B-cell clones. It would be collective characteristics of a group and is more difficult to determine than the mean life of a single clone.

Motivation to develop our mathematical model further comes also from experimental and clinical medicine and from the progress of microarray technologies.

Hampe (10) reviewed the role of anti-idiotypic antibodies in autoimmunity, including Type 1 Diabetes. There is experimental evidence of anti-Id mediated neutralization of autoantibodies, e.g., in Myasthenia gravis, or suppression of autoantibody secretion, e.g., in Idiopathic thrombocytopenic purpura. For a number of autoimmune diseases including systemic lupus erythematosus and autoimmune thyroid diseases it has been shown that anti-Id specific to autoantibodies are present in patients during remission and/or in healthy individuals, whereas it is absent during periods of active disease. The formation of anti-Id-autoantibody complexes makes it difficult to detect the single constituents by conventional assays, but several methods have been developed to overcome this problem.

Monoclonal antibodies become rapidly important in clinical therapies of autoimmune and inflammatory diseases, see e.g., Ref. (67). This gives a strong motivation to improve our understanding of systemic consequences of immunomanipulation.

The rapid technological progress makes large scale studies of the expressed idiotypic repertoire feasible. The use of antigen microarrays to profile the autoantibody repertoire in health and disease is reviewed in Ref. (68), for an application of network theory to detect antibody trees associated with antigen see (69). Immunosignaturing, reviewed in Ref. (70) uses random-sequence peptide microarrays. Microarrays using antibodies or proteins are however still expensive and complicated (70). In addition, inferring the network architecture from a sample, which is only a snapshot of a subset of the expressed idiotypic repertoire is a very demanding task.

Our model, which provides an analytical understanding of the network architecture, could be helpful to formulate conditions for a new generation of experiments with the aim to infer the networks architecture and to elucidate its role in healthy conditions and disease. From the viewpoint of statistical physics or systems biology, the question appears natural and most interesting whether there is a general principle which guides the evolution of the idiotypic network.

## 6 Glossary

- **Nodes:** A node of the network represents a clone of B-lymphocytes of a certain idiotype together with its antibodies. At a given time a node can be either occupied or empty, the corresponding clone is present or absent, respectively.
- **Bitstrings:** An idiotype is encoded by a bitstring of length *d* with entries 0 or 1. There is a total of 2*^d^* different bitstrings, which is the size of the potential idiotypic repertoire.
- **Links:** A link of the network connects two nodes with complementary idiotype, i.e., with complementary bitstrings. We do not require perfect complementarity but allow for up to *m* mismatches. The links represent the possible idiotypic interactions between the clones of the potential idiotypic repertoire.
- **Base graph:** The base graph consists of all nodes and their links for a given choice of *d* and *m*. It represents the potential idiotypic repertoire and the possible idiotypic interactions, which take place if the linked nodes are occupied.
- **Influx:** The influx of new B-lymphocytes with random idiotype from the bone marrow is modeled by occupying empty nodes with a certain probability *p* in each iteration of an update procedure.
- **Window rule:** The window rule decides whether an occupied node will survive the update or not. It will only remain occupied if the number of its occupied neighbors is neither too small nor too high but lies within an allowed window with lower and upper thresholds, *t_L_* and *t_U_*. The window rule is applied in parallel for all nodes of the network in each iteration of an update.
- **Evolution:** Iterating the steps of random innovation (influx) and deterministic selection (window rule) induces an evolution which leads, after a transient period, toward a quasistationary state of the network which may have, depending on the parameter setting, a very complex architecture.
- **Architecture:** In the steady state, groups of nodes can be identified which share statistical properties such as mean occupation and mean occupation of neighbors. The number of groups, their size, and their linking remain constant and characterize the architecture. The most interesting architecture, considered in this paper, comprises a connected part (core and periphery), a hereof disconnected part (singletons), and groups of suppressed clones (stable holes).
- **Pattern modules:** The architecture can be build by arranging identical smaller units, the pattern modules which are constructed like the base graph but have a smaller dimension *d_M_*. A pattern module contains at least one node from every group. Given *d_M_*, the number of groups, their size, and their linking can be calculated.
- **Stable Holes:** Group of nodes that are mainly unoccupied because the number of their occupied neighbors typically exceeds by far the upper threshold of the window rule, therefore we call this group stable holes. The mean occupation is close to zero.
- **Core:** Groups consisting of nodes with links to nodes in the same group build the core. The mean occupation is very low.
- **Periphery:** Groups consisting of nodes linked to the core and to stable holes, but not to nodes in its own group. The core and periphery correspond to the central part of the network. The mean occupation is high.
- **Singletons:** Groups of nodes that are only connected to stable holes. An occupied singleton can survive if it has, after the influx step, an occupied neighbor (in the group of stable holes) which typically does not survive applying the window rule. The mean occupation is high.
- **Mean-field theory:** The mean-field theory allows for a given architecture to calculate statistical properties of the groups, independent of simulations. The main simplification is that the actual occupation of neighboring nodes is replaced by their mean occupation which works the better the more neighbors are involved.
- **Self:** In the extended version of the model, self is represented by permanently occupied nodes of the network that exert influence on the linked neighbor nodes but are themselves not affected by idiotypic interactions. The window rule does not apply to self nodes.

## Conflict of Interest Statement

The authors declare that the research was conducted in the absence of any commercial or financial relationships that could be construed as a potential conflict of interest.
